# Endoscopic video defogging using luminance blending

**DOI:** 10.1049/htl.2019.0095

**Published:** 2019-12-06

**Authors:** Xiongbiao Luo, Fan Yang, Hui-Qing Zeng, Yan-Ping Du

**Affiliations:** 1 School of Informatics, Xiamen University, Xiamen 361005, People's Republic of China; 2 Zhongshan Hospital, Xiamen University, Xiamen 361005, People's Republic of China

**Keywords:** image enhancement, endoscopes, medical robotics, surgery, video signal processing, medical image processing, biomedical optical imaging, image sequences, endoscopic video defogging, endoscopic video sequences, direct surgical field, robotic surgery, surgical vision, surgical operations, luminance blending framework, foggy endoscopic video processing, clinical endoscopic videos, endoscopic video images, cauterisation

## Abstract

Endoscopic video sequences provide surgeons with direct surgical field or visualisation on anatomical targets in the patient during robotic surgery. Unfortunately, these video images are unavoidably hazy or foggy to prevent surgeons from clear surgical vision due to typical surgical operations such as ablation and cauterisation during surgery. This Letter aims at removing fog or smoke on endoscopic video sequences to enhance and maintain a direct and clear visualisation of the operating field during robotic surgery. The authors propose a new luminance blending framework that integrates contrast enhancement with visibility restoration for foggy endoscopic video processing. The proposed method was validated on clinical endoscopic videos that were collected from robotic surgery. The experimental results demonstrate that their method provides a promising means to effectively remove fog or smoke on endoscopic video images. In particular, the visual quality of defogged endoscopic images was improved from 0.5088 to 0.6475.

## 1 Introduction

Interventional endoscopes (e.g. bronchoscope and colonoscope) integrated with video cameras at their distal tips are widely introduced in minimally invasive surgery. The endoscope provides surgeons with real-time endoscopic video sequences that are shown on medical displays. On the basis of endoscopic vision or surgical field from these images, surgeons can directly visualise and examine abnormal tissues and treat or resect tumours in the body.

**Fig. 1:**
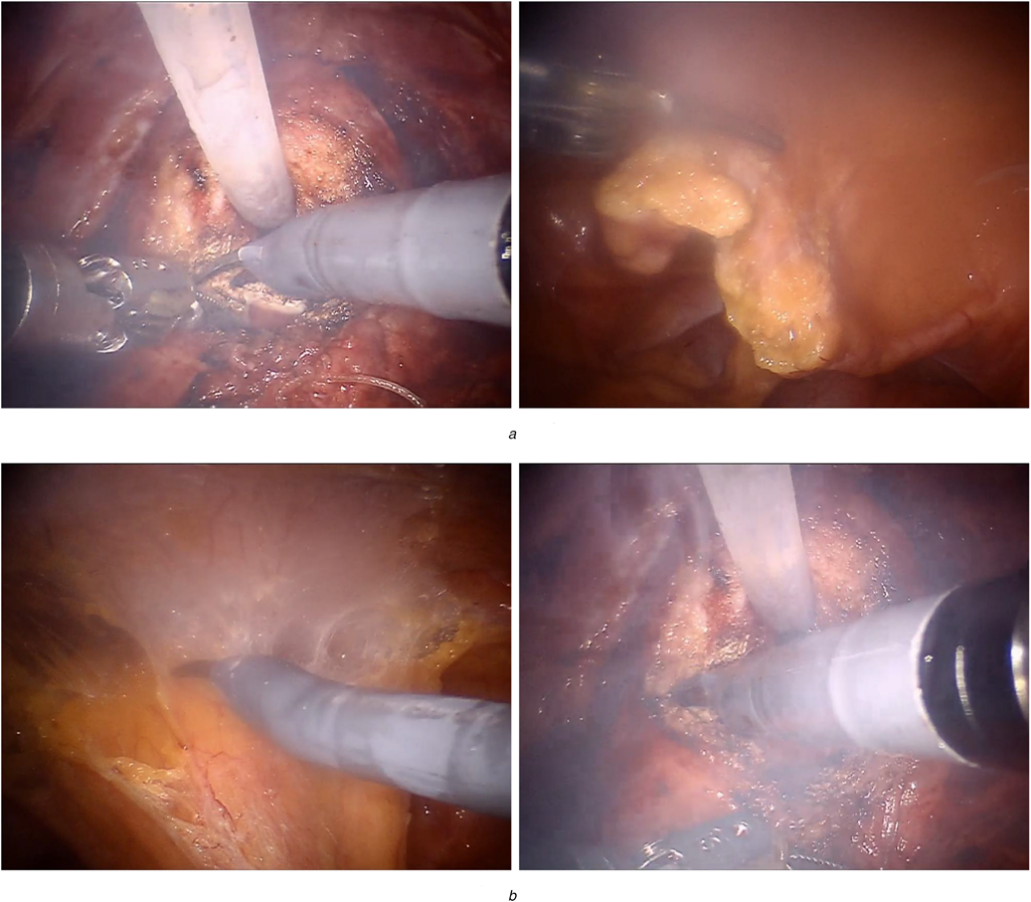
Hazy images in robotic-assisted endoscopic surgery *a* Thin smoke *b* Heavy smoke

Unfortunately, the visual quality of endoscopic video images is unavoidably degraded because of surgical smoke or fog during robotic surgery. These endoscopic foggy images (Fig. 1) are generally generated from a surgical processing called cauterisation, which is usually employed to limit the bleeding vessels, while other typical operations such as laser ablation can also bring surgical smoke in surgical field. Such fog or smoke commonly distracts surgeons who may wait for a while without doing anything until surgical smoke is gone, which increases surgical time. On the other hand, surgical fog also degrades the clear visualisation of the surgical field from the endoscope, as well as covers the structural details (e.g. vessel structures) on the organ surface. This harmful issue leads to inappropriate device use and incorrectly targeted tissue, increasing surgical risks such as in tissue or tumour resection during endoscopic surgery. Therefore, endoscopic video defogging plays an essential role in enhancing and maintaining a clear field of surgical vision, not only for safety by preventing inadvertent injury, but also for improving precision and reducing operative time.

Endoscopic field defogging methods generally consists of hardware- and software-based strategies. While the former uses typical devices to remove smoke, the latter is algorithmic, i.e. computational photography techniques. This work develops a new luminance blending strategy for surgical video defogging. It combines a contrast enhancement procedure with a fast visibility recovery method to remove fog or smoke on endoscopic video sequences. We also quantitatively and objectively evaluate the experimental results of using our proposed method and others. The main contributions of this work are two-fold: (i) a new luminance blending approach with better performance than other defogging approaches and (ii) an objective image quality metric for quantitative assessment of dehazed images.

The remainder of this Letter is organised as follows. Section 2 briefly reviews work related to current dehazing methods. Our hybrid luminance blending-based dehazing method for vision augmentation is presented in Section 3, followed by the experiment settings in Section 4. Sections 5 shows and discusses the validation results before concluding this work in Section 6.

## 2 Related work

Real-world natural image and video dehazing or defogging techniques are widely discussed in computer vision and computational photography in the literature. Fattal [1] presented a graphical model used to calculate the atmospheric light for hazy-free image recovery. They assume that scene shading and transmission are locally independent of each other, which are not practical in applications. Tarel and Hautiere [2] introduced a fast visibility restoration strategy based on median filtering, but it usually results in colour distortion and easily fails at the image median filtering step that usually introduces null pixels. On the other hand, the fast visibility method also requires more efficient computation for real-time processing.

While He *et al.* [3] proposed dark channel-based atmospheric light and transmission estimation with soft editing, Meng *et al.* [4] employed the boundary constraint and contextual regularisation to modify this dark channel-based method, especially, they improved the computational efficiency and skipped soft editing. Nishino *et al.* [5] estimated two statistically independent components of the scene albedo and depth by using the Bayesian defogging model. While this Bayesian-based method works well, it also results in colour distortion. Ancuti and Ancuti [6] discussed a multi-scale fusion approach that combines the white balance with linearly transformed images extracted from hazy images. This multi-scale fusion approach is generally trapped in dealing with inhomogeneous fog due to loss of transmission depth information. While Sulami *et al.* [7] proposed a reduced formation model to describe image pixels in small patches as lines that are used to recover the atmospheric light orientation, Galdran *et al.* [8] presented an improved variational framework using inter-channel contrast in optimisation. More interestingly, fusion-based defogging is generally recognised as a promising framework to address the disadvantages of various dehazing methods [9].

More recently, deep learning-driven methods are increasingly developed for single image dehazing. While Ren *et al.* [10] employed multi-scale convolutional neural networks for single image dehazing, Li *et al.* [11] proposed All-In-One Dehazing Network (AOD-NET) to directly create the clean image through a lightweight convolutional neural network instead of separately computing the transmission map and the atmospheric light for single image dehazing. Moreover, Ren *et al.* [12] developed a deep video dehazing method based on semantic segmentation, which can effectively use the abundant information that exists across neighbouring frames for precise dehazing. Liu *et al.* [13] introduced a simple generic model-agnostic convolutional neural network trained end-to-end to recover clear images from hazy inputs.

Although these methods work well on natural images, they remain challenging to deal with surgical endoscopic video image fog or smoke, particularly in the case of inhomogeneous or thick haze. This work aims to address the problem of hazy images or videos with inhomogeneous or thick haze, particularly foggy endoscopic videos.

## 3 Approaches

**Fig. 2:**
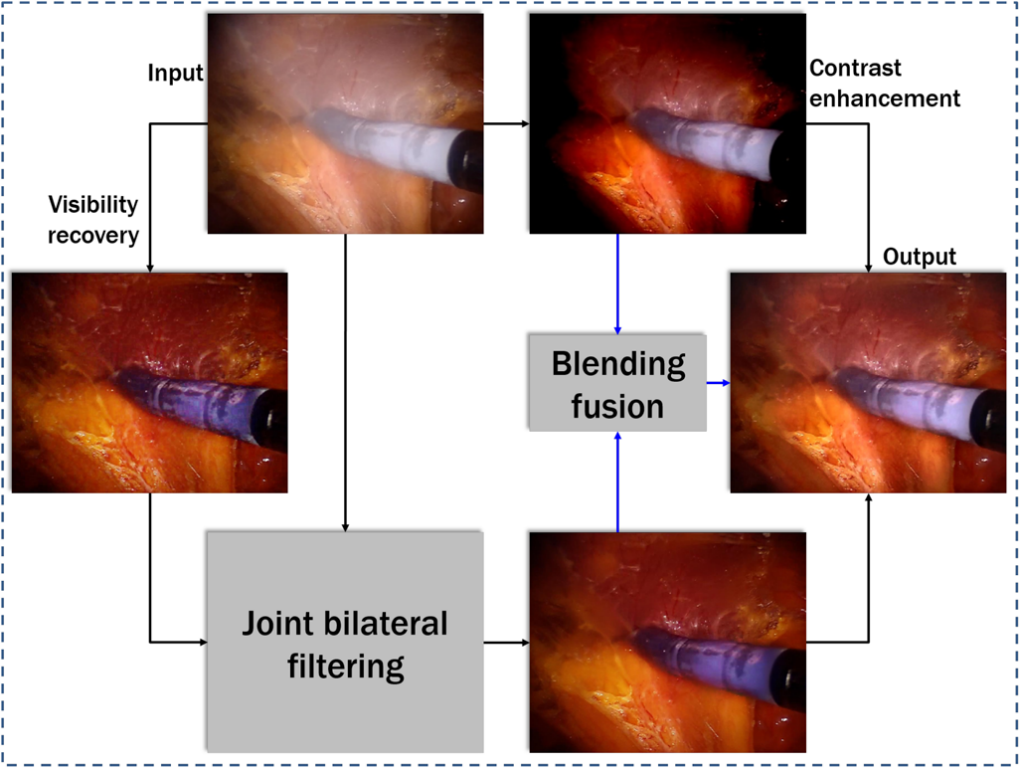
Flowchart of our proposed defogging method for night-time images

This section details our luminance blending framework for surgical endoscopic video defogging. Our method contains several steps: (i) contrast enhancement, (ii) visibility recovery, and filtering, and (iii) luminance blending. Fig. 2 shows the flowchart of our processing, as discussed in the following section.

### 3.1 Contrast enhancement

Surgical foggy images are of low-contrast and limited illumination, especially in hazy regions. The goal of contrast enhancement is to improve the contrast of hazy-less regions on the endoscopic image and calculate the luminance ${\bi L}\lpar u\comma \; \, v\rpar $ and to enhance the luminance of the final defogged surgical image.

The contrast enhancement step assumes (i) most regions on the foggy image are hazy pixels that critically affect the mean of the foggy image and (ii) the level of haze in these regions depends on the distance between the atmospheric light and the scene, as discussed in [6]. On the basis of the assumption, we compute the enhanced luminance ${\bi L}\lpar u\comma \; \, v\rpar $ by the magnifying difference between the surgical hazy image ${\bi I}\lpar u\comma \; \, v\rpar $ and its average luminance value $\lambda $ in the three channels $c \in \lcub {\rm r}\comma \; \, {\rm g}\comma \; \, {\rm b}\rcub $

(1) $${\bi L}_{\rm c}\lpar u\comma \; \, v\rpar = \beta \lpar {\bi I}_{\rm c}\lpar u\comma \; \, v\rpar - \lambda \rpar \comma \; \, \lambda = \displaystyle{{\sum\nolimits_U {\sum\nolimits_V {{\bi H}\lpar u\comma \; \, v\rpar } } } \over {UV}}\comma \; \eqno\lpar 1\rpar $$

where $\beta $ is the magnification factor to control the luminance of the augmented foggy regions and $U \times V$ are the width and height of the hazy endoscopic image. The original luminance ${\bi H}\lpar u\comma \; \, v\rpar $ at each pixel is calculated by [14]

(2) $${\bi H}\lpar u\comma \; \, v\rpar = a \times {\bi I}_{\rm r}\lpar u\comma \; \, v\rpar + b \times {\bi I}_{\rm g}\lpar u\comma \; \, v\rpar + c \times {\bi I}_{\rm b}\lpar u\comma \; \, v\rpar \comma \; \eqno\lpar 2\rpar $$

where coefficients $a = 0.299$, $b = 0.587$, and $c = 0.114$.

### 3.2 Visibility recovery and filtering

A widely used physical imaging model is established for hazy images by Koschmieder's law [3]

(3) $${\bi I}\lpar u\comma \; \, v\rpar = {\bi F}\lpar u\comma \; \, v\rpar {\bi T}\lpar u\comma \; \, v\rpar + {\bi A}_\infty \lpar 1 - {\bi T}\lpar u\comma \; \, v\rpar \rpar \comma \; \eqno\lpar 3\rpar $$

where ${\bi I}\lpar u\comma \; \, v\rpar $ denotes an observed (foggy) image, ${\bi F}\lpar u\comma \; \, v\rpar $ refers to as a haze-free image (also called scene radiance), and ${\bi A}_\infty $ indicates the atmospheric light or the sky luminance. The transmission map ${\bi T}\lpar u\comma \; \, v\rpar $ describes the amount of the unscattered light entering a camera and can be computed by

(4) $${\bi T}\lpar u\comma \; \, v\rpar = \exp \lpar - kd\lpar u\comma \; \, v\rpar \rpar \eqno\lpar 4\rpar $$

where *k* and $d\lpar u\comma \; \, v\rpar $ are the atmosphere's scattering factor and the distance between the camera and any objects in a scene.

On the basis of (3), we aim to solve hazy-free image ${\bi F}\lpar u\comma \; \; v\rpar $ under the unknown variables ${\bi A}_\infty $ and ${\bi T}\lpar u\comma \; \; v\rpar $. However, according to a fast visibility recovery method [2], we did not directly estimate ${\bi T}\lpar u\comma \; \; v\rpar $ since it is difficult to precisely predict the transmission map related to depth information. To skip ${\bi T}\lpar u\comma \; \, v\rpar $, the atmospheric veil ${\bi X}\lpar u\comma \; \, v\rpar $ was employed [6]

(5) $${\bi X}\lpar u\comma \; \, v\rpar = {\bi A}_\infty \lpar 1 - {\bi T}\lpar u\comma \; \, v\rpar \rpar \comma \; \, {\bi T}\lpar u\comma \; \, v\rpar = 1 - \displaystyle{{{\bi X}\lpar u\comma \; \, v\rpar } \over {{\bi A}_\infty }}.\eqno\lpar 5\rpar $$

Then, (3) can be rewritten to calculate ${\bi F}\lpar u\comma \; \, v\rpar $

(6) $${\bi F}\lpar u\comma \; \, v\rpar = \displaystyle{{{\bi A}_\infty \lpar {\bi I}\lpar u\comma \; \, v\rpar - {\bi X}\lpar u\comma \; \, v\rpar \rpar } \over {{\bi A}_\infty - {\bi X}\lpar u\comma \; \, v\rpar }}.\eqno\lpar 6\rpar $$

This requires the atmospheric light ${\bi A}_\infty $ and veil ${\bi X}\lpar u\comma \; \, v\rpar $ for which robust estimates can be obtained much more easily than the depth and transmission maps in the original formulation (3). The methods that are used to determine ${\bi A}_\infty $ and veil ${\bi X}\lpar u\comma \; \, v\rpar $ have been discussed in [2]. Here, we skip the technical details of how to estimate light ${\bi A}_\infty $ and veil ${\bi X}\lpar u\comma \; \, v\rpar $.

Since the result ${\bi F}\lpar u\comma \; \, v\rpar $ of the fast visibility recovery usually contains image noise and artefacts, we employ joint bilateral filtering to process ${\bi F}\lpar u\comma \; \, v\rpar $ and obtain ${\bi J}\lpar u\comma \; \, v\rpar $.

The bilateral filter is an edge-aware image processing method to denoise and simultaneously preserve edge information [15, 16]. The concept of joint bilateral filtering is to perform spatial filtering (particularly a Gaussian kernel) on a low-resolution image and simultaneously apply a range filter to process a high-resolution image (here the low- and high-resolution images refer to the recovery image ${\bi F}\lpar u\comma \; \, v\rpar $ and the original image ${\bi I}\lpar u\comma \; \, v\rpar $, respectively) [17]

(7) $${\bi J}\lpar {\bi p}\rpar = \displaystyle{1 \over {K_p}}\sum\limits_{{\bi q} \in N_p} {\bi F}\lpar {\bi q}\rpar \Omega \lpar {\bi p}\comma \; \, {\bi q}\rpar \comma \; \eqno\lpar 7\rpar $$

(8) $$\Omega \lpar {\bi p}\comma \; \, {\bi q}\rpar = \exp \left({\displaystyle{{{\left\Vert {{\bi p} - {\bi q}} \right\Vert }^2} \over {2\sigma _{\rm s}^2 }} + \displaystyle{{{\left\Vert {{\bi I}_{\rm u}\lpar {\bi p}\rpar - {\bi I}_{\rm u}\lpar {\bi q}\rpar } \right\Vert }^2} \over {2\sigma _{\rm c}^2 }}} \right)\eqno\lpar 8\rpar $$

where ${\bi p} = \lpar u\comma \; \, v\rpar $, ${\bi q} = \lpar \hat u\comma \; \, \hat v\rpar $, variances $\sigma _{\rm s}$, $\sigma _{\rm c}$ in the region $N_p$ centred at the pixel ${\bi p}$, and $K_p$ is computed by

(9) $$K_p = \sum\limits_{{\bi q} \in N_p} \exp \left({\displaystyle{{{\left\Vert {{\bi p} - {\bi q}} \right\Vert }^2} \over {2\sigma _{\rm s}^2 }} + \displaystyle{{{\left\Vert {{\bi I}_{\rm u}\lpar {\bi p}\rpar - {\bi I}_{\rm u}\lpar {\bi q}\rpar } \right\Vert }^2} \over {2\sigma _{\rm c}^2 }}} \right)\comma \; \eqno\lpar 9\rpar $$

which is the normalisation term to guarantee the sum of the weights for all the pixels to be one.

### 3.3 Luminance blending

This step is to estimate illumination on image ${\bi J}\lpar u\comma \; \, v\rpar $ and ${\bi L}\lpar u\comma \; \, v\rpar $ and blend their illumination to improve the illumination of the defogged endoscopic surgical image.

We transfer the images ${\bi J}\lpar u\comma \; \, v\rpar $ and ${\bi L}\lpar u\comma \; \, v\rpar $ from the red, green and blue (RGB) to YCbCr colour space. For the *Y*-component or luminance component of them, we used recursive filtering [18] to estimate the illumination of ${\bi J}\lpar u\comma \; \, v\rpar $ and ${\bi L}\lpar u\comma \; \, v\rpar $ and obtain ${\bi G}_J\lpar u\comma \; \, v\rpar $ and ${\bi G}_L\lpar u\comma \; \, v\rpar $. By using image illumination ${\bi G}_J\lpar u\comma \; \, v\rpar $ and ${\bi G}_L\lpar u\comma \; \, v\rpar $, we seek to recognise pixels in hazy regions. So, a weight function ${\bi W}_K\lpar {\bi G}_K\lpar u\comma \; \, v\rpar \rpar \comma \; \, K \in \lcub J\comma \; \, L\rcub $ is empirically introduced, and the output ${\bi O}_e\lpar u\comma \; \, v\rpar \comma \; \, e \in \lcub {\rm Y}\comma \; \, {\rm Cb}\comma \; \, {\rm Cr}\rcub $ of the blending fusion can be formulated

(10) $${\bi O}_e\lpar u\comma \; \, v\rpar = \displaystyle{{\sum\nolimits_{K \in \lcub J\comma \, L\rcub } {{\bi W}_K\lpar {\bi G}_K\lpar u\comma \; \, v\rpar \rpar {\bi O}_e\lpar u\comma \; \, v\rpar } } \over {\sum\nolimits_{K \in \lcub J\comma \, L\rcub } {{\bi W}_K\lpar {\bi G}_K\lpar u\comma \; \, v\rpar \rpar } }}.\eqno\lpar 10\rpar $$

Note that the weight function ${\bi W}_K\lpar{\cdot}\rpar $ (also called the weight matrix) depends on the level of smoke. In the heavy-smoke case, if the foggy pixel intensity belongs to the range of 16–128, these pixels will be assigned with weight 1. In the thin-smoke case, the pixels on the interval [128, 235] will be assigned with weight 1. The luminance output ${\bi O}_Y\lpar u\comma \; \, v\rpar $ may not be distributed into the full range of pixel intensity, resulting in a low-contrast image. We implement the following linear transformation to stretch its histogram to a specific intensity range $\lsqb P\comma \; \, Q\rsqb $

(11) $${\hat{\bi O}}_Y\lpar u\comma \; \, v\rpar = P + \displaystyle{{{\bi O}_Y\lpar u\comma \; \, v\rpar - {\bi O}_{{\rm Min}}\lpar u\comma \; \, v\rpar } \over {{\bi O}_{{\rm Max}}\lpar u\comma \; \, v\rpar - {\bi O}_{{\rm Min}}\lpar u\comma \; \, v\rpar }}\lpar Q - P\rpar \comma \; \eqno\lpar 11\rpar $$

where ${\hat{\bi O}}_Y\lpar u\comma \; \, v\rpar $ denotes the final luminance, ${\bi O}_{{\rm Min}}\lpar u\comma \; \, v\rpar $ and ${\bi O}_{{\rm Max}}\lpar u\comma \; \, v\rpar $ are the minimum and maximum intensities of the blending output ${\bi O}_Y\lpar u\comma \; \, v\rpar $, respectively. We empirically set $P = 15$ and $Q = 236$ in our work. Eventually, we combine the *Y*-component ${\hat{\bi O}}_Y\lpar u\comma \; \, v\rpar $ and the chromatic components ${\bi O}_{{\rm Cb}}\lpar u\comma \; \, v\rpar $ and ${\bi O}_{{\rm Cr}}\lpar u\comma \; \, v\rpar $ and transform them into the RGB colour space, obtaining the final defogged image.

## 4 Validation

Foggy endoscopic videos were acquired from robotic surgery. All the experiments were executed on a laptop installed with Windows 8.1 Professional 64 bit system, 32.0 GB memory, and processor Intel(R) Xeon(R) CPU $ \times $ 8 2.8 GHz and MATLAB R2017a. We tested about 1200 frames in this Letter.

We compare the proposed method with the following approaches: (i) M1, Tarel *et al.* [2], (ii) M2, He *et al.* [3], (iii) M3, Nishino *et al.* [5], (iv) M4, Ancuti and Ancuti [6], (v) M5, Meng *et al.* [4], (vi) M6, Sulami *et al.* [7], and (vii) M7, our method.

We introduce a naturalness metric to depict how natural surgical images appear based on statistically analysing thousands of images [19]. On the other hand, we also employ structural similarity index (SSIM) [20] to evaluate structural information on images. Eventually, we define a hybrid quality metric $\psi $ to evaluate defogged endoscopic images

(12) $$\psi = \gamma {\cal S} + \lpar 1 - \gamma \rpar {\cal N}\comma \; \eqno\lpar 12\rpar $$

where ${\cal S}$ denotes the SSIM and ${\cal N}$ indicates the naturalness. The coefficient $\gamma $ is set to 0.6, which was experimentally determined to balance the structural information and naturalness.

## 5 Results and discussion

**Fig. 3:**
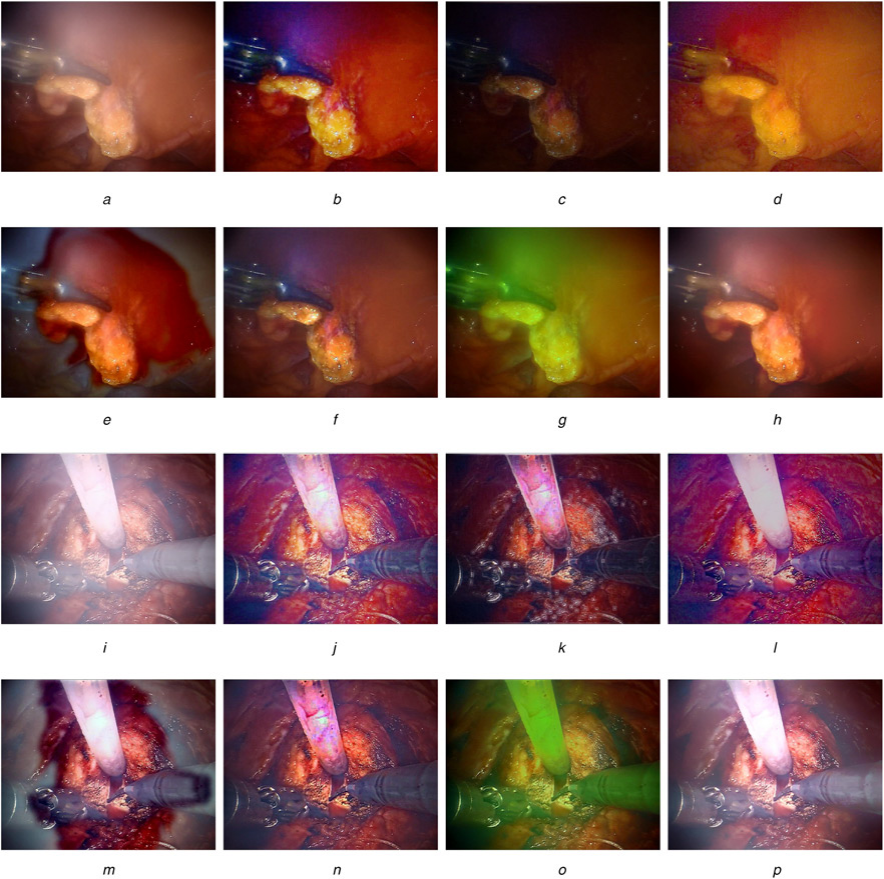
Comparison of using various defogging methods: (a)–(h) thin-fog image and (i)–(p) thick-fog image *a* Thin-fog image *b* M1 [2] *c* M2 [3] *d* M3 [5] *e* M4 [6] *f* M5 [4] *g* M6 [7] *h* M7 (ours) *i* Thick-fog image *j* M1 [2] *k* M2 [3] *l* M3 [5] *m* M4 [6] *n* M5 [4] *o* M6 [7] *p* M7 (ours)

Fig. 3 visually compares the defogged results of endoscopic images with thin and thick fogs. The visual quality of the results demonstrates that our method works better than others since it removes fog without introducing colour distortion. On the other hand, two surgeons manually inspected all the dehazed results and generally believe that our defogged method outperforms other approaches since the subjective visual quality of using our method is more natural and colourful, which is better than the original foggy image.

Table 1 quantitatively compares the objective assessment of the dehazed results obtained from the seven approaches. The quantitative assessment results show that our proposed approach outperforms other methods. While the average naturalness of M1 and our proposed method M7 were comparable, the average SSIM was improved from 0.7978 to 0.9275. More interestingly, the average hybrid quality of our methods was 0.6475, which was much better than other approaches (M5 provides 0.5088).

**Table 1:** Quantitatively objective assessment of the results obtained from the seven defogging approaches

Approaches	M0	M1 [2]	M2 [3]	M3 [5]	M4 [6]	M5 [4]	M6 [7]	M7 (ours)
SSIM ${\cal S}$	—	0.6587	0.4781	0.6676	0.6488	0.7978	0.3944	0.9275
naturalness ${\cal N}$	0.1411	0.2319	0.0218	0.1097	0.1439	0.0752	0.0602	0.2274
hybrid $\psi $	—	0.4890	0.2956	0.4445	0.4468	0.5088	0.2608	0.6475

M0 indicates the quantitative results of the original foggy images and does not have the SSIM index that is a reference-based metric

Additionally, the computational times of the methods M1, M3, M4, M5, M6, and M7 were 31.3, 62.3, 1.3, 5.2, 75.6, and 1.1 s/frame, respectively. Method M2 deals with an image in more than 2700 s since soft editing was extremely slow [3]. Our method works faster than others.

This work aims to enhance the surgical field visualisation of endoscopic surgery. We developed a new luminance blending defogging algorithm. The experimental results demonstrate that our algorithm outperforms others from subjective and objective evaluations. The effectiveness of our algorithm lies in fusing the advantages of the enhancement and restoration dehazing methods. Our method has several potential limitations including unclear parameter sensitivity, effective enhancement, quality assessment, and heavy processing time. These limitations will be further investigated in the future. In addition, though our method works better than other approaches, it still introduces colour distortion, which will be further investigated.

## 6 Conclusion

We proposed a new luminance blending defogging framework that integrates contrast enhancement, joint bilateral filtering, and visibility recovery to remove smoke in endoscopic videos from robotic surgery. We evaluated our method on endoscopic video sequences acquired from robotic prostatectomy. The experimental results demonstrate the effectiveness of our proposed method, which outperforms other approaches. In particular, our method improved the hybrid quality of the dehazed results from 0.5088 to 0.6475.
